# Monocular Depth Estimation with Self-Supervised Learning for Vineyard Unmanned Agricultural Vehicle

**DOI:** 10.3390/s22030721

**Published:** 2022-01-18

**Authors:** Xue-Zhi Cui, Quan Feng, Shu-Zhi Wang, Jian-Hua Zhang

**Affiliations:** 1 School of Mechanical and Electrical Engineering, Gansu Agriculture University, Lanzhou 730070, China; cuixuezhi504@163.com; 2 College of Electrical Engineering, Northwest University for Nationalities, Lanzhou 730030, China; dqwsz@xbmu.edu.cn; 3 Agricultural Information Institute of CAAS, Beijing 100081, China; zhangjianhua@caas.cn

**Keywords:** edge computing device, monocular depth estimation, self-supervised learning, vineyard scene

## Abstract

To find an economical solution to infer the depth of the surrounding environment of unmanned agricultural vehicles (UAV), a lightweight depth estimation model called MonoDA based on a convolutional neural network is proposed. A series of sequential frames from monocular videos are used to train the model. The model is composed of two subnetworks—the depth estimation subnetwork and the pose estimation subnetwork. The former is a modified version of U-Net that reduces the number of bridges, while the latter takes EfficientNet-B0 as its backbone network to extract the features of sequential frames and predict the pose transformation relations between the frames. The self-supervised strategy is adopted during the training, which means the depth information labels of frames are not needed. Instead, the adjacent frames in the image sequence and the reprojection relation of the pose are used to train the model. Subnetworks’ outputs (depth map and pose relation) are used to reconstruct the input frame, then a self-supervised loss between the reconstructed input and the original input is calculated. Finally, the loss is employed to update the parameters of the two subnetworks through the backward pass. Several experiments are conducted to evaluate the model’s performance, and the results show that MonoDA has competitive accuracy over the KITTI raw dataset as well as our vineyard dataset. Besides, our method also possessed the advantage of non-sensitivity to color. On the computing platform of our UAV’s environment perceptual system NVIDIA JETSON TX2, the model could run at 18.92 FPS. To sum up, our approach provides an economical solution for depth estimation by using monocular cameras, which achieves a good trade-off between accuracy and speed and can be used as a novel auxiliary depth detection paradigm for UAVs.

## 1. Introduction

Depth information has been proved extremely useful in various computer vision and robotic tasks, and it is also one of the essential research aspects in the field of unmanned agriculture vehicles (UAV) [1]. Researchers have developed a variety of depth detection sensors to obtain depth data, such as infrared distance sensor [2], ultrasonic distance sensor [3], laser distance sensor [4]. In recent years, higher-performance depth detectors, such as binocular cameras [5,6,7,8,9], millimeter-wave radar [10,11] and lidar [12,13,14,15,16] have also been equipped on UAVs to complete relevant missions. Researchers also found that the accuracy of depth information can be effectively improved by using several depth detectors simultaneously [17]. According to this discovery, an environment perceptual system combining lidar and cameras is designed for our UAV. A lidar is mounted on the UAV’s top as the primary depth detector while several cameras are mounted around the body, which provides information on both object detection and additional depth information, especially the blind area of the lidar. As we all know, in China, the farmers’ choices are susceptible to the price of agricultural machinery. In our design, the following approaches are considered to reduce the cost: Firstly, thanks to algorithm advance of monocular depth estimation, cheaper monocular cameras are adopted instead of traditional binocular cameras. Secondly, NVIDIA’s edge computing device—JETSON TX2 is chosen as the computing platform to carry out the depth estimation mission. This paradigm combines the advantages of cheapness, compact size and high energy efficiency. 

In the past many years, the method of depth detection with lidar has been studied extensively [12,13,14,15,16] while estimating depth information from a single image taken by a monocular camera is attracting more research interest [18,19,20,21,22,23,24,25,26,27,28,29,30,31,32,33,34,35,36,37,38,39,40,41,42,43]. Monocular depth estimation is essentially vague and a technically ill-posed problem: With only an image, there will be an infinite number of possible world scenes where the image comes from. Therefore, it is much more complicated to obtain depth data from monocular images using general digital image processing technology based on geometric principles than binocular cameras. The difficulty of monocular depth estimation has attracted considerable attention for over a decade, and researchers have developed many methods to complete the task. Generally, their methods can be categorized into two kinds: methods based on hand-crafted features and probabilistic graphical models [18,19,20,21,22], and methods using convolutional neural networks (CNN) [23,24,25,26,27,28,29,30,31,32,33,34,35,36,37,38,39,40,41,42,43]. 

At the early stage, monocular depth estimation always depended on hand-crafted features and probabilistic graphical models. For example, Saxena et al. [18] estimated distinct image patches’ absolute scales and got depth relation from a Markov Random Field model. Non-parametric ways [19,20,21,22] were also proposed to infer the depth of a query image with the method of combining the depths of images with similar photometric content retrieved from a database.

In recent years, researchers have been making critical achievements to improve the performance of CNN models on many visual tasks, such as classification [44,45,46,47,48], and object detection [49,50]. The advantages of CNN also motivated researchers to use it in the tasks of pixel-level prediction, e.g., segmentation [51,52]. Specifically, when provided with an extensive training set of pairs of images and corresponding depth maps, depth estimation can be regarded as a pixel-level regression problem. CNN models can be trained to directly infer the depth value associated with every pixel of an RGB image. Due to its paramount application value, monocular depth estimation methods based on CNN models have become a hot topic. Compared with hand-crafted methods, CNN-based methods significantly improve in accuracy and robustness. These methods can be further divided into the supervised method and the self-supervised method. 

The supervised method [23,24,25,26,27,28,29] requires plenty of image samples annotated with ground truth depth labels to get expected results. Eigen et al. [23] combined two different scales of CNN to carry out the monocular depth estimation, in which the global coarse-scale network predicts the coarse depth relation and the local fine-scale network infers the details of the global coarse-scale network. Scale-invariant loss function updates the parameters. Xu et al. [24], proposed a depth model using continuous conditional random fields to fuse the complementary information output by multiple CNN sub-networks. The model cascades multiple continuous conditional random fields at a specific scale to realize multi-scale feature fusion, thus achieving more effective depth prediction. Fu et al. [25] discretized depth and recast depth network learning as a regression problem with a strategy called spacing-increasing discretization (SID). They used a multi-scale network to simplify their structure while capturing more feature details. They trained their model (DORN) with ordinary regression loss. Thus, DORN got excellent performance over the KITTI dataset. Li et al. [26] combined a deep convolutional neural network (DCNN) and a conditional random field (CRF) to estimate the depth of a single image. The former was engaged to extract depth features, the latter was used to refine the details. The combination received a competitive performance over the Make3D dataset. Cao et al. [27] regarded monocular depth estimation tasks as a pixelwise classification task. They first discretized the continuous actual depth into several spans and marked them with the depth ranges. Then, a fully convolutional deep residual network was trained to solve the classification problem. The supervised approach needs lots of time to make the training dataset annotated with detailed depth information. 

The self-supervised method (also known as the unsupervised method) is also a popular strategy for depth estimation [30,31,32,33,34,35,36,37,38,39,40,41,42,43] which usually uses monocular image sequences in video streams as training sets and geometric constraints of the sequences are based on projections between adjacent frames. This method does not mean that a network is trained without any supervised information, it means the algorithm can automatically obtain the supervised relation between the unlabeled data and the data generated during the training period. Therefore, the self-supervised method is more efficient than the supervised approach. Grag et al. [30] made an automatic encoder to achieve self-supervised monocular depth estimation. In training, the convolutional encoder was trained by applying stereo pair images (source and target) to predict the depth map of the source image. The inverse distortion of the target image is generated explicitly using the predicted depth and the known inter-view displacement is used to reconstruct the source image, in which the photometric error in reconstruction is the reconstruction loss of encoder. Godard et al. [31] reduced the requirement of factual depth information with self-supervised learning architecture and Left-Right Consistency in the MonoDepth network model. The disparity maps of the left eye and the right eye are inferred by RGB images passing through the left eye of a binocular camera. Then the corresponding images of the left eye and the right eye are reconstructed by combination (for example, the right eye image is reconstructed using the left-eye image and the right disparity map). The consistency between the reconstructed image and the original image is detected to achieve the self-supervised purpose. MonoDepth2 [43], a self-supervised monocular depth estimation algorithm proposed by Godard et al. after MonoDepth, is realized by calculating the loss between the input image and the reconstructed input image by using networks’ results comprehensively, which enhances the overall efficiency. Yang et al. [34] formulated an edge-aware depth-normal consistency term and estimated the depth of a single image by constructing a depth-to-normal layer and a normal-to-depth layer inside of the deep convolutional network (DCN) they proposed. That is a method that implemented self-supervised training by comparing the depth estimated and the depth output from normal. Mahjourian et al. [36] estimated the depth with CNN and proposed a novel self-supervised strategy that considers the consistency of the estimated 3D point clouds and the ego-motion across successive frames. Besides Mahjourian et al., Liu et al. [37] also proposed a self-supervised novel method, the Attention-Pixel and Attention-Channel Network (APAC-Net), for self-supervised monocular learning of estimating scene depth and ego-motion. The Temporal-consistency loss LTemp between adjacent frames and the Scale-based loss LScale among different scales were utilized to train the network. Yin et al. [38] proposed GeoNet which has three modules: estimate monocular depth, optical flow and ego-motion. The network uses the geometric relations extracted from the three different modules to determine an image reconstruction loss, reasoning about static and dynamic scene parts separately.

In this paper, a CNN-based model called MonoDA is proposed to estimate depth which can be run on our perceptual system of UAV and infer the depth information from video taken by monocular cameras. The model is designed as a lightweight model to run on the edge computing device with limited resources. Furthermore, we introduce the self-supervision method to simplify the labeling and training process. Image sequences from monocular video streams are employed to train the model. The model has two subnetworks: depth estimation and pose estimation subnetwork. The former deriving from U-Net, is used to infer depth maps, and the latter extracts images’ features and outputs the pose transformation relation between two adjacent images in a clip of video stream. Our main contributions are two-fold:

(i) A lightweight monocular depth estimation model which has competitive accuracy and lower color sensitivity.
(ii) A novel depth estimation scoring method that comprehensively considers major evaluation metrics of depth estimation and models’ performance on NVIDIA JETSON TX2.

The experimental results show that our perceptual system and the depth estimation model are appropriate for agriculture scenarios (like vineyards). The remainder of this paper is organized as follows. Section 2 presents the architecture and training method of the MonoDA. Section 3 describes the requirements and evaluation metrics of several experiments. Section 4 depicts the experimental results and discussion. Section 5 concludes the paper.

## 2. Methods

### 2.1. The Structure of MonoDA

**Figure 1:**
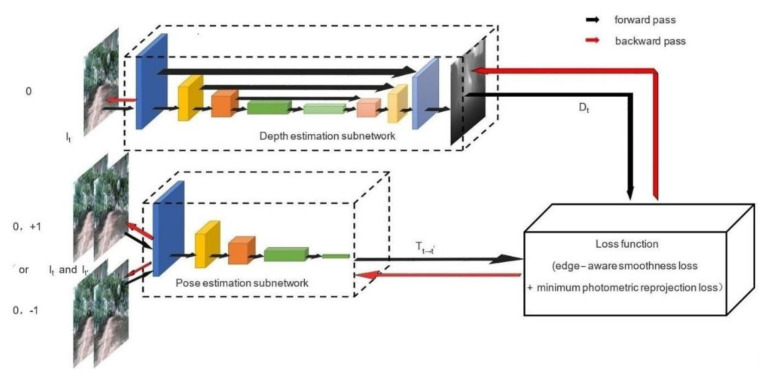
The structure of MonoDA.

MonoDA, a monocular depth estimation model based on a self-supervised method, is built to use monocular frame sequences in video streams in training. The model comprises two subnetworks, one is the depth estimation subnetwork, and the other is the pose estimation subnetwork. The function of the depth estimation subnetwork is to infer a depth map from the RGB input image (current frame) through the forward pass of the network. The forward pass result of the pose estimation subnetwork is a transformation matrix obtained from two adjacent frames of the input video. In the training process, we reconstruct the current input image by depth map and the matrix, calculate the loss between the input image and the reconstructed image, and update the two subnetworks’ parameters through the backward pass. The structure is shown in Figure 1.

In Figure 1, 0, −1, +1 represent the current, the last, and the next frame of a monocular video respectively. $I_{t}$, $I_{t^{’}}$ is the input image and the adjacent frame of input respectively. $T_{t\to t^{’}}$ refers to the Inter-frame transformation matrix. $D_{t}$ indicates the depth map inferred. In the loss function module, the reconstructed input is first obtained, and then the loss function is calculated between the reconstructed image and the input.

### 2.2. Depth Estimation Subnetwork

**Figure 2:**
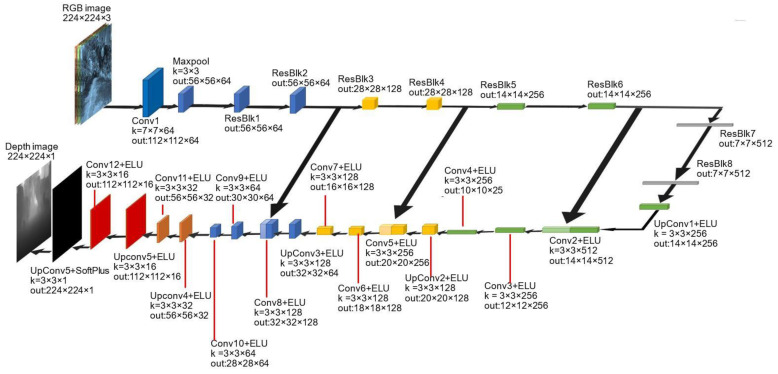
The depth estimation subnetwork.

U-Net [53] is a typical structure for pixel-wise prediction of depth estimation [54,55]. Following this trend, this structure is also employed to create MonoDA’s depth estimation subnetwork. As shown in Figure 2, our depth estimation subnetwork is a modified version of U-Net consisting of an encoder, a decoder, and three bridges. The encoder gradually extracts depth features of different sizes, and ResNet18 [47] is utilized to build each convolution block. The function of the decoder is to restore the feature map output by the encoder to the size of the input and infer depth pixel by pixel, as shown at the bottom of Figure 2. In our decoder, the depth map with the size of the input is generated by six up-convolution structures (up-sampling + convolution). The bridges are used to add the depth information from the shallow layers of the encoder to the deep layers of the decoder, making the contours of objects in the depth map clearer. To be specific, some feature maps extracted from the encoder’s layers will be copied, resized, and stacked on the corresponding decoder’s layers. The whole structure of the subnetwork is presented in Figure 2.

In Figure 2, Convx (x∈[1, 12], x∈N^+^) means Convolution block. K = a×b×c means length×width×channel of convolution kernel. out: w×h×z means the size of the output feature map. ResBlkx (x∈[1, 8], x∈N^+^) means 8 residual blocks of ResNet18, and the structure detail is shown in [47]. Maxpool, UpConvx (x∈[1, 5], x∈N^+^), ELU and SoftPlus represent maximum pooling layer, up-convolution block, ELU activation function and SoftPlus activation function respectively. The size of the rectangular block is the output feature maps’ size. The rectangular blocks with the same color mean that they have the same number of channels, and the lighter ones are copied from the darker ones with the same color after size modification. 

**Figure 3:**
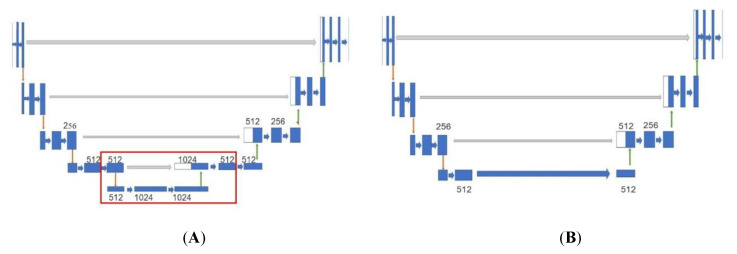
Part structures of U-Net and depth estimation subnetwork. (**A**) U-Net; (**B**) Depth estimation subnetwork of MonoDA.

As shown in Figure 3, the structure of U-Net is simplified as follows: remove the feature layers and operations related to 1024 channels (convolution, maximum pooling, up-convolution, and bridge), reduce the calculation amount and model size by processing the 512-channel feature layer with the computation and connection mode of the 1024-channel layer. Subsequent experiments show that this method speeds up depth estimation effectively. For the prediction layer, the last two layers are replaced with our inference structure where the SoftPlus activation function is used instead of the Sigmoid activation function, which is used widely in other depth estimation models. The expression of the SoftPlus activation function is shown as follows:

(1) $$\mathrm{SoftPlus}={\log(1+e}^{x})$$

The following experimental results establish that this way makes depth inference more conform to actual scenes. 

Figure 3A is U-Net [53], in which the red box is the part we want to modify. Figure 3B is our modified structure. The numbers in the figure indicate the numbers of the channels. Blue, red, green, and gray arrows represent convolution, maximum pooling, up-convolution, and bridge operations respectively. 

### 2.3. Pose Estimation Subnetwork

**Figure 4:**
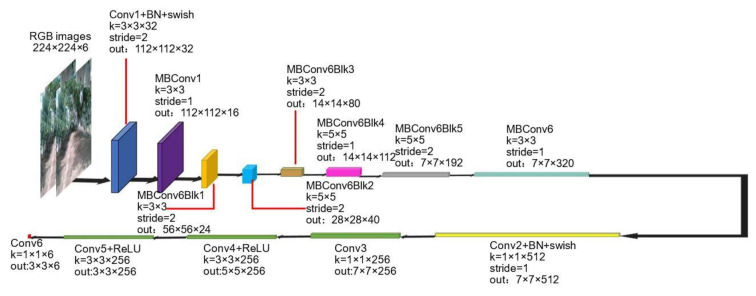
The pose estimate subnetwork.

The structure of the pose estimation subnetwork is shown in Figure 4. Two adjacent frames (224 × 224 × 3) from the input video are stacked in channel dimension to obtain an input tensor of 224 × 224 × 6. In this subnetwork, EfficientNet-B0 [56] is employed as a backbone network to extract input features. Then, the feature map with the size of 7 × 7 × 512 extracted from the backbone network will pass through four continuous convolution blocks to gain a final pose transformation matrix (3 × 3 × 6), where six channels represent the rotation matrix and translation matrix in X, Y and Z directions respectively.

In Figure 4**,** the notation of stride represents stride operation. MBconvxBlky (x = 1 or 6, y∈[1, 5], y∈N^+^) represents 7 MBconv blocks in EfficientNet-B0 [56]. Maxpool, BN, ReLU, and swish represent maximum pooling layer, batch normalization, ReLU activation function and swish activation function respectively. The other notations have the same meanings as Figure 2. The input is two adjacent frames from video stacked in channel dimension. 

### 2.4. Training Method

The loss function and training method of MonoDepth2 are used for training our model. First, the adjacent frame $I_{t^{’}}$ of current frame $I_{t}$ is re-projected to obtain $I_{t^{’}\to t}$, which is the reconstructed current frame, the formulation is as follows:

(2) $$I_{t^{’}\to t}{=I}_{t^{’}}{<\mathrm{proj}(D}_{t}{,\;T}_{t\to t^{’}},\;K)>$$

$D_{t}$ and $T_{t\to t^{’}}$ are the results of subnetworks, proj() are the resulting 2D coordinates of the projected depths $D_{t}$ in $I_{t^{’}}$, K is the internal parameters matrix of all cameras collecting the dataset, assuming that they are all known and have the same value, <> is a sampling operator. 

Then, we use the minimum photometric reprojection loss $L_{P}$ to calculate the difference between $I_{t^{’}\to t}$ and $I_{t}$. The calculation formula can be written as:

(3) $$L_{P}=\underset{t^{’}}{\min}{\mathrm{Pe}\;(I}_{t}{,\;I}_{t^{’}\to t})$$

where $\underset{t^{’}}{\min}$ means the minimum value of Pe on $I_{t^{’}}$, Pe is defined as photometric reprojection loss:

(4) $$\mathrm{Pe}\left(I_{t}{,\;I}_{t^{’}\to t}\right)=\frac{\alpha }{2}\left(1-\mathrm{SSIM}\left(I_{t}{,\;I}_{t^{’}\to t}\right)\right)+\left(1-\alpha \right){\left|{|I}_{t}{,\;I}_{t^{’}\to t}|\right|}_{1}$$

In the above formula, α is 0.85, ${\left|{|I}_{t}{,\;I}_{t^{’}\to t}|\right|}_{1}$ and $\mathrm{SSIM}\left(I_{t}{,\;I}_{t^{’}\to t}\right)$ are the expressions of calculating $L_{1}$-norm and structural similarity with${\;I}_{t}$ and $I_{t^{’}\to t}$ respectively and the latter can be written as:

(5) $$\mathrm{SSIM}\left(I_{t}{,\;I}_{t^{’}\to t}\right)=\frac{{\;(2u}_{I_{t}}u_{I_{t^{’}\to t}}{+c}_{1}{)\;(2\sigma }_{I_{t}I_{t^{’}\to t}}{+c}_{2})}{{\;(u}_{I_{t}}^{2}{+u}_{I_{t^{’}\to t}}^{2}{+c}_{1}{)\;(\sigma }_{I_{t}}^{2}{+\sigma }_{I_{t^{’}\to t}}^{2}{+c}_{2})}$$

where $u_{I_{t}}$, $u_{I_{t^{’}\to t}}$, $\sigma_{I_{t}}^{2}$ and $\sigma_{I_{t^{’}\to t}}^{2}$are the average values and the variances of all pixels involved in $I_{t}$ or $I_{t^{’}\to t}$ respectively. $\sigma_{I_{t}I_{t^{’}\to t}}$ is the covariance of all pixels involved in $I_{t}$ and $I_{t^{’}\to t}$.${\;c}_{1}\;$and $c_{2}\;$are the small normal numbers which aim at avoiding the denominator being 0. The closer the result of SSIM is to 1, the more similar the two images are. 

An automatic mask coefficient μ is added to${\;L}_{P}$ to judge the occlusion relation between objects through the change of continuous frames and the motion attributes of pixels relative to the camera, ignore which almost immobile pixels and improve the estimation speed.

(6) $$\mu =\left[\underset{t^{’}}{\min}{\mathrm{Pe}\;(I}_{t}{,\;I}_{t^{’}\to t})<\underset{t^{’}}{\min}{\mathrm{Pe}\;(I}_{t}{,\;I}_{t^{’}})\right]$$

“[]” is the Iverson bracket, which is 1 if the conditions in the brackets are met, or 0 otherwise. 

Then, $I_{t}$ is processed by using the edge-aware smoothness loss function $L_{S}$, to strengthen the learning effect of images’ edge and eliminate the edge noise and the expression can be defined as:

(7) $$L_{s}=\left|\partial_{x}d_{t}^{*}\right|e^{-|\partial_{x}I_{t}|}+\left|\partial_{y}d_{t}^{*}\right|e^{-|\partial_{y}I_{t}|}$$

Where $d_{t}^{*}=\frac{d_{t}}{{\bar{d}}_{t}}$ is the average normalized inverse depth aiming at preventing the shrinkage of the estimated depth.

Finally, the total loss function L is shown in Formula (8):

(8) $${L=\mu L}_{P}{+\lambda L}_{S}$$

In the above formula, the λ is the smoothing coefficient and the value is 0.001. 

Adam optimization algorithm is used to update the parameters of the model.

## 3. Materials and Experiments

### 3.1. Platforms and Software Environments

The training platform is equipped with Intel^®^ CORETMi5-8400 six-core CPU(Intel Corp.; Santa Clara, CA, USA), with a basic frequency of 2.80 GHz and a maximum frequency of 4.00 GHz. NVIDIA RTX 2060 6 GB(NVIDIA Corp.; Santa Clara, CA, USA) with a basic frequency of 1210 MHz and a maximum frequency of 1435 MHz is chosen as GPU. RAMs are two Samsung DDR4@2666 MHz 8 GB(Samsung Electronics Co., Ltd; Gyeonggi do, South Korea). The operating system is Windows 10 1909 64-bit home edition(Microsoft Corp.; Redmond, DC, USA). The development environment includes NVIDIA Display Driver v460.79(NVIDIA Corp.; Santa Clara, CA, USA), CUDA Toolkit 10.2(NVIDIA Corp.; Santa Clara, CA, USA), Python3.7(Python Software Foundation; Delaware, USA), and Pytorch1.4(Facebook Inc.; Menlo Park, CA, USA). 

After training, the model was transferred to our environment perceptual system for testing. The system’s platform is edge computing device NVIDIA JETSON TX2 (after this referred to as TX2), which has a six-core CPU composed of an HMP Dual Denver 2 dual-core processor(Advanced RISC Machines; Cambridge, UK) and a Quad ARM@A57 quad-core processor(Advanced RISC Machines; Cambridge, UK) with the highest frequency of 2.00 GHz. The GPU adopts NVIDIA Tegra X2 with 256 CUDA computing cores and the highest frequency is 1300 MHz. RAM is LPDDR4 8 GB. Ubuntu18.04(Canonical Group Ltd.; London, UK), Pytorch1.5(Facebook Inc.; Menlo Park, CA, USA), Python 3.6(Python Software Foundation; Delaware, USA), CUDA Toolkit 10.0(NVIDIA Corp.; Santa Clara, CA, USA) and CUDNN 7.5.0(NVIDIA Corp.; Santa Clara, CA, USA) were installed and configured as software.

The experiments proceeded with a batch size of 6, 20 epochs, an initial learning rate of 0.0001, dataset segmentation with Eigen style [23] (the train set has 39,810 RGB images, and the test set has 4424 images), 0.1 m as minimum distance and 100 m as maximum distance.

### 3.2. Platforms and Software Environments

The KITTI raw dataset (after this referred to as Kitti) was engaged as a training dataset. The dataset was collected and designed mainly for the road scene. Two RGB cameras, two grayscale cameras, and a Velodyne 64-line lidar were adopted as data acquisition devices. The dataset has 175 GB of data, including RGB images, grayscale images, and point cloud data collected in five days (26, 28, 29, 30 September and 3 October 2011).

**Figure 5:**
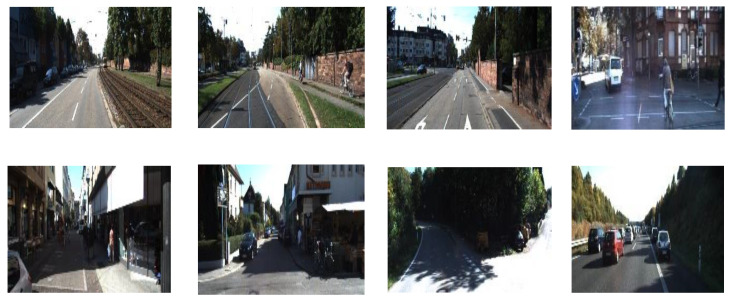
Partial images of Kitti.

As shown in Figure 5, the scene of images in Kitti is quite different from the vineyard. To evaluate the estimation accuracy of the environment perceptual system of our UAV in the actual scene of a vineyard, we established a test dataset of vineyard scenes. The Bulldog UAV manufactured by Yikun Electric Engineering Co., Ltd (Shanghai, China). was installed with the Intel RealSense D415 depth camera to capture field images in Gansu Wine Industry Technology Research and Development Center’s vineyard, Gansu Agricultural University, Lanzhou, Gansu, China. D415 is a programmable camera with one RGB camera, one Infrared structured light projector and two stereo receivers. We wrote a program with the SDK provided by Intel to mobilize these above devices of D415 in order to collect images as well as depth information. The RGB camera was used to obtain monocular images for the surrounding environment around several ridges in the vineyard. These images were utilized as inputs of models to infer the depth maps. To get depth information, firstly, the closest and the farthest detection distances of the D415 were set to 0.1 m and 100 m respectively. Then, we can obtain the farthest distance among the calculated distances of all pixels in the current image. Finally, the farthest distance was used to normalize all pixels’ distances to obtain the ground truths of depth and these truths formed the depth matrix. The depth matrix was employed as one of the evaluation metrics inputs to calculate the difference with the depth matrix converted from corresponding depth maps. Images and depth information were collected according to different periods (morning, noon, evening), weather condition (sunny, cloudy, rainy) and illumination (no shadow, a little shadow, a lot of shadows, forward light, and backlight). We ultimately got 500 images and matrices.

### 3.3. Experiments and Evaluation Metrics

#### 3.3.1. Model Evaluation Metrics

In order to evaluate the accuracy of models over Kitti or our vineyard dataset, five widely used evaluation metrics in the field of monocular depth estimation were selected. They were absolute relative error (Abs Rel), square relative error (Sq Rel), root mean square error (RMSE), logarithmic root mean square error (LG RMSE) and accuracy (Accuracy, %). The calculated formulas are as follows:

(9) $$\mathrm{Abs}\;\mathrm{Rel}=\frac{1}{N}\sum_{i=1}^{N}\frac{{|D}_{i}{-D}_{i}^{*}|}{D_{i}^{*}}$$

(10) $$\mathrm{Sq}\;\mathrm{Rel}=\frac{1}{N}\overset{N}{\underset{i=1}{\sum }}\frac{{|D}_{i}{-D}_{i}^{*}{|}^{2}}{D_{i}^{*}}$$

(11) $$\mathrm{RMSE}=\sqrt{\frac{1}{N}\overset{N}{\underset{i=1}{\sum }}{|D}_{i}{-D}_{i}^{*}{|}^{2}}$$

(12) $$\mathrm{LG}\;\mathrm{RMSE}=\sqrt{\frac{1}{N}\overset{N}{\underset{i=1}{\sum }}{|\mathrm{lgD}}_{i}{-\mathrm{lgD}}_{i}^{*}{|}^{2}}$$

(13) $$\;\mathrm{Accuracy}:\;\max\left(\frac{D_{i}}{D_{i}^{*}},\;\frac{D_{i}^{*}}{D_{i}}\right)=\delta \;<\;T$$

In Formulas (9)–(13), N, D_i_, D_i_^*^ and T represent the total number of image pixels, the estimation depth value of the ith pixel, the ground truth of depth corresponding to the ith pixel and three different thresholds (1.25, 1.25^2^, 1.25^3^) respectively. The smaller the values of Formula (9) to Formula (12) are, the fewer the errors of the depth estimation model occur and the better the estimation effect is. The larger the value of Formula (13) is, the higher the accuracy of the depth estimation model is.

#### 3.3.2. Real Distance Test and Evaluation

The depth value represents relative distance relation, and it is an 8-bit floating-point value ranging from 0 to 1. The bigger the depth value is, the farther the distance represents, which means that the depth matrix converted from the depth map is not the actual distances; it is just a matrix demonstrating the depth relation. Therefore, if we want to measure the actual distance with the model, the matrix’s elements need to be converted to actual distances. As mentioned above, the minimum and maximum distances were set to 0.1 m and 100 m respectively in the training period, which means the element value 0 and 1 of the matrix correspond to 0.1 m or less and 100 m or more respectively. Reverse inferring can be obtained according to the normalized expression of depth value provided in [41]; the estimated distance can be defined as:

(14) $$\mathrm{Dis}=\frac{(100-0.1)\mathrm{Dep}}{1-0.1\mathrm{Dep}}$$

where Dep and Dis represent the original element in a depth matrix and the estimated distance respectively.

We designed an experiment to determine whether the estimated distance met the actual demand. Firstly, D415 was set up at ‘0 m’ position and a plate sign was placed directly in front of D415 within 10 m so that the actual distance between D415 and the sign could be recorded accurately. Then, the image taken by D415 was fed into MonoDA to get a depth map converted to distance by the Formula (14). Finally, from the image, the average distance of the sign and we calculated the relative error with the accurate distance. We placed the sign at various distances to compute the average of relative errors. The relative error is calculated by Formula (15).

(15) $$R=\frac{\mathrm{eDis}-\mathrm{relDis}}{\mathrm{relDis}}\times 100\%$$

In the formula above, eDis and relDis represent the estimated distance and the real distance respectively. The domains of relDis are (0,10) and R, where 0 and 10 respectively represent the minimum and maximum distance between the two respectively in the experiment. The closer this value is to 0, the better the result is.

#### 3.3.3. Model Comprehensive Test and Scoring Method on TX2

Running the real-time depth estimation program on TX2 will occupy a large number of system resources, which may affect the performance of other programs (such as object detection) of the perceptual system of UAV. We hope to choose the optimal one from several comparison models to provide a trade-off among different requirements. So taking accuracy, speed, and hardware resource occupancy into account, we suggested a scoring method to evaluate the comprehensive performance of real-time depth map inference of vineyard scenes for different models. 

The score can be computed as follows:

(16) $$w_{\mathrm{ij}}\;=\frac{w_{i}}{n_{i}}$$

(17) $$S_{T}=\overset{3}{\underset{i=1}{\sum }}\sum_{j=1}^{n_{i}}w_{\mathrm{ij}}s_{\mathrm{ij}}$$

In Formula (16), i (i = {1, 2, 3}) represents the three main items which are ‘Depth Estimation Effect (DEE, i = 1)’, ‘Average of Frames Per Second (AFPS, i = 2)’ and ‘Hardware Resource Occupation (HRO, i = 3)’ respectively. $w_{i}$ represents the weights of the above three main items defined respectively and the sum of which is 1; n_i_ indicates the number of subitems of the ith item and j (j = {1, 2, …, n_i_}) represents its jth subitem. $w_{\mathrm{ij}}$ represents the weight of item ‘ij’. The detail refers to Table 1. For example, DEE concludes three subitems EI, AI, and ARE, which reflect the accuracy of a model from different aspects. Each subitem is divided into five levels according to the experimental results. When a subitem’s value falls into the specific range stipulated in Table 1, we assign the corresponding score from 1 to 5. In detail, we give 5 for the best value of a subitem among the models. When the value drops 20%, the score is reduced by 1. In Formula (17), $S_{T}$ is the total score and $s_{\mathrm{ij}}$ means the score of subitem ‘ij’.

HRO contains three subitems: GPU usage, CPU usage and RAM usage, which indicates the model’s situation of hardware occupancy. The scoring method is similar to DEE. 

To measure the speed and hardware occupancy, TX2 was set to maximum performance mode with all irrelevant terminals closed in our experiment. Jetson-Stats of TX2 was employed to monitor a model’s program and record the average values of subitems within 30 s.

From the practical experience of the UAV operation, the speed is of the most importance, DEE comes second, and HRO last. Therefore, we assign the weights of items following their importance degree, among which w1 (DEE) is 0.4, w2 (AFPS) is 0.5, and w3 (HRO) is 0.1.

## 4. Results

To assess the performance of our method, several experiments in various settings were conducted. We give quantity results over Kitti, as well as comparing results with several famous monocular depth estimation methods. The model was also evaluated over a vineyard dataset taken in an actual work scene. Further, we estimated real distance by our model. Finally, we provided the comprehensive experiments’ results of our model on TX2.

### 4.1. Accuracy Evaluation Results 

We have selected some monocular depth estimation models [38,39,40,41,42,43] based on the self-supervised method for comparative experiments with our method. For a fair principle, the super parameters and split strategy (Eigen split [23], mentioned in Section 3.1) of training for all the models were the same. Table 2 reveals the results of Kitti. In this table, the smaller the Error items, the higher the accuracy of the model. On the contrary, the bigger Accuracy items, the higher the accuracy of the model. The italic numbers are the best results and the underlined numbers are the suboptimum results(the meanings of italic numbers and underlined numbers in other tables are same as Table 2’s). According to this criterion, it can be seen from Table 2 that MonoDepth2 is ahead of other methods in all metrics, and our model ranks 2nd best on 6 out of 7 metrics. To sum up, MonoDepth2 and MonoDA are obviously superior to the others.

In order to reduce the complexity of experiments, in the rest of this paper, we only provided the results of the best two models in Table 2, namely MonoDepth2 and MonoDA, for further experiments.

The vineyard dataset was used to assess the model’s performance in the real work scene for UAVs. The evaluation results are shown in Table 3. Compared with the results over Kitti, the results over the vineyard dataset are significantly different: their values of Error items become worse to some extent while the values of Accuracy items become better. Furthermore, the positions of the two are reversed: MonoDA has 8 out of 9 best results while MonoDepth2 only has one. That means the accuracy of MonoDA surpassed MonoDepth2 as a whole. However, the gap between the values of the two sets is very small. The evidence above reveals that in the vineyard scene, our model can get a better estimation effect.

In order to assess the actual distance performances of the two models, an experiment was implemented. In our experiment setting, the plate sign was placed at nine different positions within 10 m. We derived the estimated distances and compared them with actual distances to analyze the accuracy of the natural scene. The results are shown in Table 4. The first line of the distance item in Table 4 is the shortest distance between the signs and the camera, the second and third lines correspond to the average value in the sign’s area estimated by the two models and converted from Formula (14). It can be seen that the distances estimated of the two models are close to the actual distances, our method gets 4 best out of 9 while MonoDepth2 gets 5 best out of 9. However, ARE of MonoDA is slightly better than MonoDepth2. This indicates that the accuracy of distance estimation of the two methods is totally equivalent. The 14% of ARE is acceptable for the application of UAV.

### 4.2. Model Comprehensive Test Results

The real-time depth estimation results are shown in Table 5, the scores of comprehensive test results calculated according to the scoring standard in Table 1 are shown in Table 6. It is worth saying that the scores of subitems of DEE were calculated by Table 3 and Table 4. The scores of DEE of the models—MonoDepth2 is 2.7 and MonoDA is 3.0, indicating our model’s accuracy was higher than MonoDepth2 in the vineyard scene.

The average inference speed of MonoDepth2 is 16.84 FPS and scores 2.0 while MonoDA is 18.92 FPS and scores 3.0, which indicates that our model also performed better than MonoDepth2. 

As for resource occupation, MonoDepth2’s average occupation rate of GPU is 51.92%, which occupies a high amount of GPU computing resources. However, this model has lower requirements for CPU computing power: TX2 6 cores are not fully loaded, the second (24~78%) and third (26~76%) cores were mainly used to estimate depth. In terms of RAM occupation, 1.8 GB is occupied. The score of MonoDepth2 is 3.0.

MonoDA’s average occupancy rate of GPU is 45.42%, which is 6.5% lower than MonoDepth2. For our model’s CPU occupancy, TX2 six cores are not fully loaded either. Nevertheless, compared with MonoDepth2, the occupancies of all cores are slightly increased. In terms of RAM occupation, MonoDA has the same occupancy of 1.8 GB. The score of MonoDA is 4.0. To conclude, our model’s resource occupation is lower than MonoDepth2. This result attributes to our simpler network architecture.

According to Formula (17) and the data in Table 5, the final scores of the two models are shown in Table 6: MonoDA was 3.1, and MonoDepth2 was 2.4. The results indicate that MonoDA has better all-around performance and is more suitable for real-time depth estimation in the vineyard.

## 5. Discussion

In Table 2, the models for comparison [38,40,41,42,43] adopt the depth and pose estimation subnetworks to solve the depth estimation problem, and many of them [38,39,40,41,43] engage the encoder-decoder structure to estimate the depth relation; [38,40] both employ the ResNet50 as the backbone of the depth estimation network’s encoder, which is a network deeper than our backbone (ResNet18). Moreover, these models also use bridge structures as we do. The model of [39] employs the U-Net structure to estimate the depth relation, and so do we. However, the structure of direct visual odometry is used to estimate the pose transformation relations instead of the pose estimation network. The model of [41] uses the VGG structure as the backbone of the encoder to estimate the depth map. Moreover, it engages a holistic 3D motion parser (HMP) structure to design loss functions. The depth estimation network of [42] is similar to [43] and ours, but the pose network is changed by predicting the motion of a single object rather than the entire image. MonoDepth2 [43] and MonoDA outperform the other models remarkably. This implies a better combination of structure and training method which provides higher accuracy performance. Specifically, MonoDepth2 has a similar structure to our model, they both used U-Net structure to estimate the depth relation. However, compared with four bridges in MonoDepth2, MonoDA only has three bridges (referring to Figure 2), which reduces the information in shallow layers fused in deep layers and slightly affects the evaluation effect. Besides, the two models’ pose estimation subnetworks have a different backbone.

**Figure 6:**
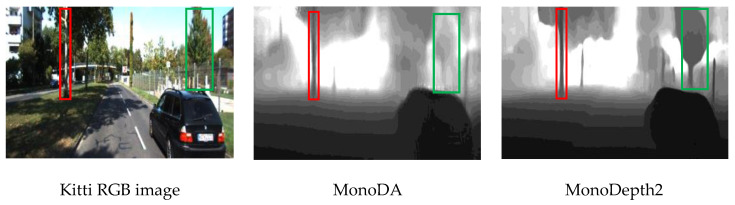
The example depth maps inferred by models with Kitti image.

**Figure 7:**
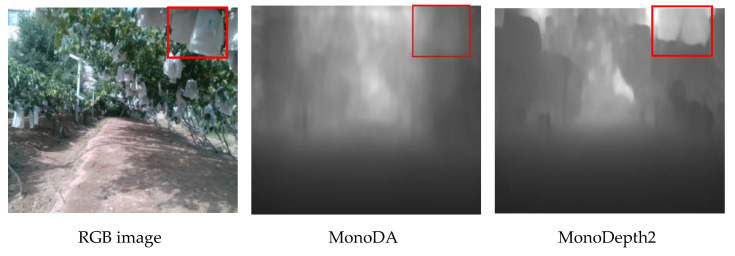
The example of a depth map of vineyard scene inferred by the models.

In addition to the statistical results given in Table 2, we observe some subtle differences between the top two models through some examples. As shown in Figure 6 and Figure 7, whatever in the Kitti scene or the vineyard scene, the depth maps inferred by MonoDepth2 have more clear contours in the depth map. However, MonoDepth2 may produce inappropriate depth relation among different objects while our model performs better in this respect. For example, in Figure 6, the tree in the green box is farther than the one in the red box, but the depths of the two trees inferred by MonoDepth2 are almost the same under two situations, which is inconsistent with the fact. However, we observe that MonoDA seems to output the proper depth relation (the green box shows lighter gray in the middle of Figure 6). Although the reduction of the bridge in MonoDA makes the counters of the depth map somewhat blur compared with MonoDepth2, the SoftPlus activation function improves the hierarchical relation of the depth map and makes the distance relation of objects in the depth map more accurate. We speculate that it is because SoftPlus is an unsaturated activation function and its change trend is gentler.

The other example is shown on the left of Figure 7. There are many small and crowded leaves in a vineyard image, which leads the feature domain of vineyard images quite different from Kitti. More complex textures made the depth map inferred by the models appear more blurry than Kitti. As shown in Figure 7, the distance between the camera and the bagging on fruit (white plastic bags) is very close, so the gray of the red box area in the depth map should be darker. However, the depth relation is miscalculated obviously by MonoDepth2 due to its sensitivity to color, so the model gives the bags lighter gray (the right of Figure 7). On the contrary, our model gives the bags dark gray (the middle of Figure 7) to depict the depth relation, which is more suitable for the actual situation. Compared with MonoDepth2, the result shows that our method is not sensitive to color changes.

According to the actual distance test results shown in Table 4, the Average Relative Error of our model is smaller than MonoDepth2, which implies our method’s performance is more stable.

We conducted experiments to test the comprehensive performance of the two models on our UAV’s computing platform TX2. According to the experimental results of Table 5 and Table 6, our method has faster speed and lower resource occupation that can be attributed to the more straightforward structure in MonoDA, which relieves the pressure of GPU.

On the whole, our method has more advantages, that is, on the premise of simplifying the model, the accuracy is almost the same, but the calculation speed and the resource occupation are significantly improved, which is more suitable for our UAV’s platform working in the vineyard scene.

## 6. Conclusions

In this paper, a lightweight self-supervised CNN model is proposed as an economical method to estimate the distances of obstacles around the UAV, which can predict a depth map from a single RGB method. The MonoDA model can be trained with frame sequences from monocular video streams. The nucleus of the model mainly consists of two subnetworks: depth estimation subnetwork and pose estimation subnetwork; the former employs an encoder-decoder structure to infer a depth map from a single image, and the latter is used to get the geometric relation between two adjacent frames of the video. Two subnetworks’ outputs are employed to reconstruct the current frame of the video, which is used to calculate the loss with the current frame and then train the model. Our training method is self-supervised, which can significantly reduce the workload of labeling.

A vineyard dataset is established. Several experiments were designed based on this dataset to test our model to assess the performance of our model in the vineyard scene. The results of our experiments prove that our model has good performance on our perceptual systems’ platform—edge computing device TX2 and is appropriate for the vineyard. Compared with the famous MonoDepth2, MonoDA has higher scores on all items tested which means MonoDA has better depth estimation accuracy, faster inference speed, and lower hardware resource occupation.

As we all know, UAVs can greatly liberate peasants, and an efficient environment perceptual system can improve the UAV’s efficiency. Therefore, as one of the critical members of the system, our distance detection method with low hardware requirements can help to promote the efficiency of the whole system and be a valuable way to popularize UAVs.

## Figures and Tables

**Table 1:** Evaluation criteria of test items over vineyard dataset.

$\mathrm{Score}\;(s_{\mathrm{ij}})$	DEE ^1^			AFPS ^2^	HRO ^3^		
	EI ^4^	AI ^5^	ARE ^6^		A1 ^7^	A2 ^8^	A3 ^9^
5	≤1.5	1.50<	0~5%	30.0<	≤30.0%	cores full ≤ 1	≤1.5 GB
4	1.5~1.625	1.35~1.50	5~10%	24.0~30.0	30.0~36.0%	2 cores full	1.5 GB~1.8 GB
3	1.625~1.750	1.20~1.35	10~15%	18.0~24.0	36.0~42.0%	3 cores full	1.8 GB~2.1 GB
2	1.750~1.875	1.05~1.20	15~20%	12.0~18.0	42.0~48.0%	4 cores full	2.1 GB~2.4 GB
1	1.875~2.00	0.90~1.05	20~25%	6.0~12.0	48.0~54.0%	5 cores full	2.4 GB~2.7 GB
0	2.00<	<0.90	25%<	<6.0	54.0%<	6 cores full	2.7 GB<

^1^ DEE, Depth Estimation Effect. ^2^ AFPS, Average of Frames Per Second. ^3^ HRO, Hardware Resource Occupation. ^4^ EI, Error Items. The value of EI is the average value of Abs Rel, Sq Rel, RMSE and LG RMSE of the model evaluated over the vineyard dataset. ^5^ AI, Accuracy Items. The value of AI is the average value of Accuracy items with three thresholds (δ < 1.25, δ < 1.25^2^ and δ < 1.25^3^) of the model evaluated over vineyard dataset. ^6^ ARE, Average Relative Error. ^7^ A1 is the usage of GPU, AVE means the average usage of GPU. ^8^ A2 is the usage of CPU, “Core full” means core occupancy ≥95%. ^9^ A3 is the usage of RAM.

**Table 2:** The evaluation results of several monocular depth estimate models over Kitti.

Model	Error items				Accuracy items		
	Abs Rel	Sq Rel	RMSE	LG RMSE	δ < 1.25	${\delta \;<\;1.25}^{2}$	${\delta \;<\;1.25}^{3}$
GeoNet [38]	0.149	1.060	5.567	0.226	0.796	0.935	0.975
DDVO [39]	0.151	1.257	5.583	0.228	0.810	0.936	0.974
DF-Net [40]	0.150	1.124	5.507	0.223	0.806	0.933	0.973
EPC++ [41]	0.141	1.029	5.350	0.216	0.816	0.941	0.976
Struct2depth [42]	0.141	1.026	5.291	0.215	0.816	0.945	0.979
MonoDepth2 NP ^1^	0.132	1.044	5.142	0.210	0.845	0.948	0.977
MonoDA (our)	0.126	1.035	5.105	0.203	0.857	0.955	0.979
MonoDepth2 [43]	*0.115*	*0.903*	*4.863*	*0.193*	*0.877*	*0.959*	*0.981*

^1^ NP, No Pretrained, means without using ImageNet pretrained model.

**Table 3:** The evaluation result of MonoDepth2 and MonoDA in vineyard scene.

Model	Error Items					Accuracy Items			
	Abs Rel	Sq Rel	RMSE	LG RMSE	Average ^1^	δ < 1.25	${\delta \;<\;1.25}^{2}$	${\delta \;<\;1.25}^{3}$	Average
MonoDA	*0.134*	1.083	*5.306*	*0.214*	*1.684*	*1.118*	*1.271*	*1.453*	*1.281*
MonoDepth2	0.144	*1.075*	5.415	0.232	1.717	1.113	1.243	1.375	1.244

^1^ Average means the average value of the sum of subitems under item.

**Table 4:** Results of estimated distance and comparison with actual distances.

Model	Distance/m									ARE ^1^
	1.60	2.40	3.20	4.00	4.80	6.40	7.20	8.00	8.80	
MonoDepth2	*1.81*	*2.07*	2.84	*3.31*	2.74	4.92	*7.29*	*8.24*	8.62	17%
MonoDA	1.34	1.94	*3.51*	3.03	*3.25*	*5.42*	6.85	8.45	*8.93*	*14%*

^1^ ARE, Average Relative Error, is the average of relative errors of 9 positions.

**Table 5:** Results of real-time depth estimation on TX2.

Model	Detection of Real-Time Video					Comprehensive Evaluation
	Average Frame Rate	A1 ^1^	A2 ^2^		A3 ^3^	
MonoDepth2	16.84	51.92%	c1:4–20% c3:26–76% c5: 2–24%	c2:24–78% c4:5–16% c6:3–16%	1.8 GB	Medium speed High GPU occupied Low CPU occupied
MonoDA	18.92	45.42%	c1:6–27% c3:32–82% c5:4–25%	c2:26–78% c4:5–24% c6:3–22%	1.8 GB	Relatively smooth Medium GPU occupied Low CPU occupied

^1^ A1 is the average usage of GPU. ^2^ A2 is the usage of CPU, ci ($i\;\in \;[1,\;6],\;i\;\in {\;N}^{+})$ means the 6 cores of CPU. ^3^ A3 is the usage of RAM.

**Table 6:** The scores of the two models.

Model	Items Score							Total Score
	$\mathrm{DEE}\;(w_{1}=0.4)$			$\mathrm{AFPS}\;(w_{2}=0.5)$	$\mathrm{HRO}\;(w_{3\;}=0.1)$			
	$\mathrm{EI}{\;}^{1}\;(w_{11}=0.133)$	$\mathrm{AI}{\;}^{2}\;(w_{12}=0.133)$	$\mathrm{ARE}{\;}^{3}\;(w_{13}=0.133)$		$A1\;(w_{31}=0.033)$	$A2\;(w_{32}=0.033)$	$A3\;(w_{33}=0.033)$	
MonoDepth2	3	3	2	2	1	5	4	2.4
MonoDA	3	3	3	3	2	5	4	3.1

^1^ EI, Error Items. The value of EI is the average value of Abs Rel, Sq Rel, RMSE and LG RMSE of the model evaluated over the vineyard dataset. ^2^ AI, Accuracy Items. The value of AI is the average value of Accuracy items with 3 thresholds (δ < 1.25, δ < 1.25^2^ and δ < 1.25^3^) of the model evaluated over the vineyard dataset. ^3^ ARE, Average Relative Error. $w_{\mathrm{ij}}{=w}_{i}{/n}_{i}$, $n_{i}$ is the number of subitems under ith item. A1, A2 and A3 are the usages of GPU, CPU and RAM respectively.

## Data Availability

Restrictions apply to the availability of these data. Data was obtained from Gansu Agriculture University.
